# MEMS-Switched Triangular and U-Shaped Band-Stop Resonators for K-Band Operation

**DOI:** 10.3390/s23198339

**Published:** 2023-10-09

**Authors:** Romolo Marcelli, Giovanni Maria Sardi, Emanuela Proietti, Giovanni Capoccia, Jacopo Iannacci, Girolamo Tagliapietra, Flavio Giacomozzi

**Affiliations:** 1 Institute for Microelectronics and Microsystems (CNR-IMM), 00133 Roma, Italy; giovannimaria.sardi@cnr.it (G.M.S.); emanuela.proietti@cnr.it (E.P.); giovanni.capoccia@cnr.it (G.C.); 2 Fondazione Bruno Kessler, Povo, 38123 Trento, Italy; iannacci@fbk.eu (J.I.); gtagliapietra@fbk.eu (G.T.); giaco@fbk.eu (F.G.)

**Keywords:** Sierpinski triangle, U-shaped resonators, frequency tunability, RF MEMS, metamaterials

## Abstract

Triangular resonators re-shaped into Sierpinski geometry and U-shaped resonators were designed, linking them with single-pole-double-through (SPDT) RF MEMS switches to provide frequency tuning for potential applications in the K-Band. Prototypes of band-stop narrowband filters working around 20 GHz and 26 GHz, interesting for RADAR and satellite communications, were studied in a coplanar waveguide (CPW) configuration, and the tuning was obtained by switching between two paths of the devices loaded with different resonators. As a result, dual-band operation or fine-tuning could be obtained depending on the choice of the resonator, acting as a building block. The studied filters belong to the more general group of devices inspired by a metamaterial design.

## 1. Introduction

Multi-band systems have begun to be widely adopted in modern satellite communication [1], where complex arrangements of frequencies and spatial coverage constraints require miniaturization [2,3] and frequency band selection. RF MEMS switches have been used for the last two decades to tune or reconfigure high-frequency devices instead of pin diodes or transistors to overcome their intrinsic limitations using an all-passive solution. Despite the obvious advantages of using microelectromechanical systems, a fully reliable and long-lifetime solution has not yet been certified for the space environment. On the other hand, a few examples [4,5,6,7,8] used in signal routing have also been studied in the past years [9,10,11] and, more recently, also for the small satellites domain [12,13,14]. For this reason, the RF MEMS solution is still appealing to substitute semiconductor conventional switches or even bulky waveguide switches with planar, miniaturized, and fully passive devices. The ongoing interest in researching RF MEMS is also devoted to RF applications towards reconfigurability; for instance, the frequency agility of resonators [15,16] and antennas [17,18]. A new perspective for the feasible application of RF MEMS is opening up with regard to 5G/6G telecommunication technologies [19,20,21], where the lowest power consumption will be a key aspect [22,23].

In this paper, RF MEMS devices will be used in a single-pole-double-through (SPDT) configuration to address the microwave signal in two branches loaded by resonators working at frequencies very close to each other (fine-tuning around 20 GHz or 26 GHz) or to switch the signal between approx. 20 GHz and 26 GHz.

The general structure of the proposed devices was designed in a coplanar waveguide (CPW) configuration for possible implementation within staked modules, i.e., in situations where the electromagnetic interference between different layers must be minimized. CPWs naturally fulfill this requirement, because the electric field is confined in the plane of the substrate. Due to their robustness and reliability, double-clamped metal beams are the best mechanical structure for RF MEMS switches. Manufacturing micromechanical devices using lateral ground planes for supporting the entire structure is convenient from a mechanical and electrical standpoint, because it is easier to separate the RF and DC paths, as generally required by high-frequency sub-systems.

Two geometries were considered in this contribution: (i) triangular resonators with a Sierpinski geometry and (ii) U-shaped resonators.

Sierpinski structures were introduced for a few reasons. First, triangular geometries are rarely used, but have some advantages regarding their footprint and coupling possibilities, as discussed in [24], concerning square or circular resonators. Secondly, there is no systematic work on or a straightforward analytical approach to determining the spectrum of Sierpinski triangles depending on their internal complexity, and an experimental approach supporting or modifying the theory is necessary for CPW-fed configurations.

This paper also studied the U-shape, combining two resonators with SPDT RF MEMS devices. In this case, we selected this configuration for miniaturization purposes and the implementation in coupled structures analogously to the classical hairpin filters developed in a microstrip configuration [25].

So far, the novelty of this basic work is mainly related to introducing the design of triangular and U-shaped resonators in a non-conventional configuration hosted by a CPW environment and driven by RF MEMS switches.

Results about the design, manufacturing, and testing of devices mounting RF MEMS SPDT switches will be presented for applications in K-Band, specifically working around 20 GHz and 26 GHz. The technology for realizing switches in MEMS technology has already been discussed in [24] and will be briefly reviewed here. In this contribution, we will discuss the advantages and drawbacks of dual-frequency and fine-tuning operations using switched resonators implemented by RF MEMS technology. Electromagnetic high-frequency simulations are performed using the Cadence AWR Microwave Office package V22.1, using the Axiem^®^ solver (Cadence Design Systems, Dublin, Ireland). The paper is structured by briefly describing the SPDT switch in Section 2, which allows for signal routing through the paths where resonators are hosted, and the signal filtering is performed. Section 3 and Section 4 describe, in parallel, the operation of the triangular- (Sierpinski) and U-shaped resonators, respectively. A conclusion about the presented work is then elaborated in Section 5.

## 2. SPDT Design, Technology, and Test

**Figure 1:**
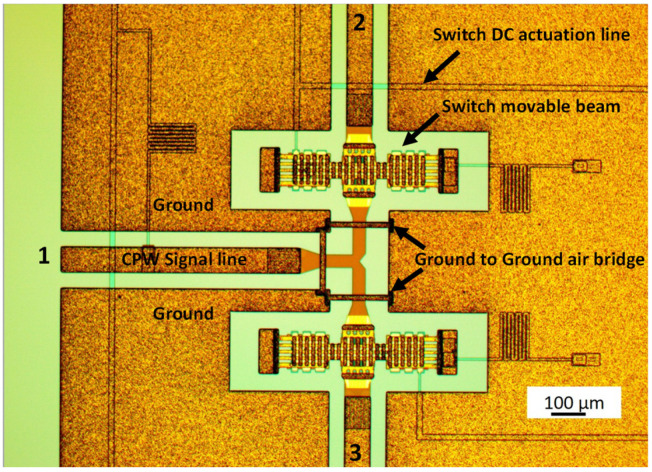
Manufactured SPDT switch. The arrows point to the actual details of the device, including the air bridges’ location with the optimized geometry of the T-junction, the individual switches, the actuation lines, and the CPW feeding line.

Two preliminary technological runs were performed to select the more promising configuration for the SPDT switch. The final layout used in this work for the actual devices is shown in Figure 1.

The MEMS switch actuation mechanism is based on electrostatically actuated fixed–fixed metal bridges suspended over the center conductors of CPW transmission lines. An air gap separates the bridge from the center conductor when no bias is applied. When a voltage is applied between these two metals, the electrostatic force starts to pull the bridge towards the lower conductor. The bias is then increased until a threshold voltage is reached, and, at this point, the bridge’s spring force is overcome; the bridge snaps down and contacts the bottom signal line.

Two clamped–clamped microswitches are used to send the input signal from port 1 to port 2 or 3. The switch beam is made of gold with a thickness of about 1.8 µm for the flexible and deformable arms and about 5 µm for the rigid main body. The switches are electrostatically actuated using high-resistivity polysilicon electrodes underneath the lateral side of the beams. The air gap between the non-actuated switch, the underneath actuation electrodes, and the bottom signal line is 3 µm.

The main improvements between the first and the second run were the introduction of three air bridges, also pointed out in Figure 1, and a new configuration of the T-section, where the lines were tapered to provide a better impedance matching of the overall device.

**Figure 2:**
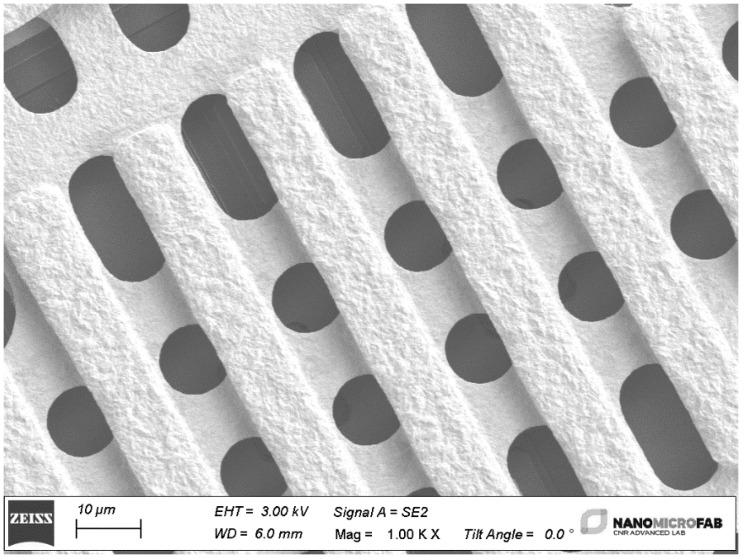
SEM micrograph with detail of the metal beam layout. In this section, the metal beam is reinforced with a double-step electroplating process to strengthen the central part mechanically, closing the interrupted central conductor of the CPW. In contrast, only a single-step electroplating is performed on the anchors to maintain an elastic response during the actuation. As usual, the holes facilitate removing the sacrificial layer underneath the bridge.

A section of the metal beam is shown in the SEM (scanning electron microscope) micrograph in Figure 2, with particular attention on the holes necessary for removing the sacrificial layer below the bridge.

The technology fabricating the entire device differed only for the MEMS switch compared to the planar resonators. In the case of the MEMS SPDT, the entire process encompassed a multilayer deposition of dielectric and metal layers, selective electroplating to make the beam more robust in the contact regions, and the final removal of a sacrificial layer to obtain the suspended beams. The complete procedure required an eight-mask process, as described in [24], and it was a simplified and optimized version of the SPDT presented in [25]. The resonators resulted from a more straightforward process using simple photolithography and electroplating to obtain a 5 μm thick metal to mitigate the skin depth effects.

**Figure 3:**
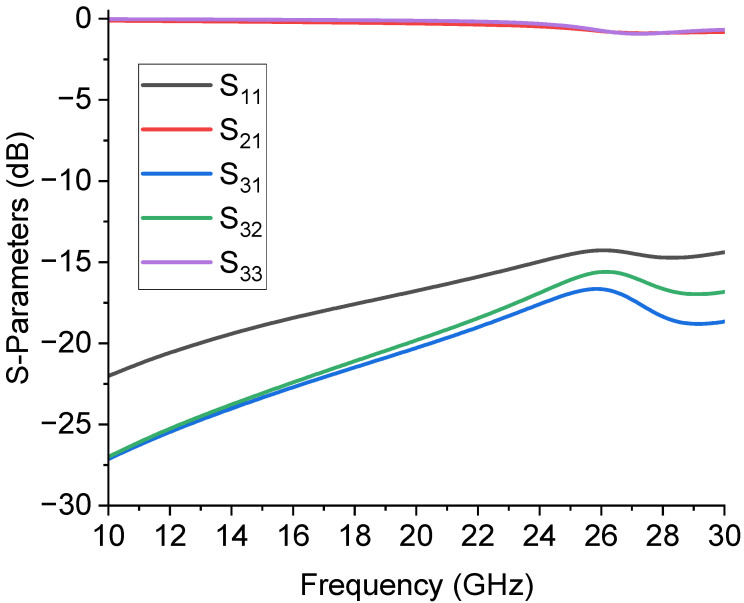
Simulated results of the SPDT configuration when the switch at port 3 is considered not actuated. Results are shown in terms of matching at the input port (S_11_), transmission to actuated branch (S_21_), isolation between ports 1 and 3 and ports 2 and 3 (S_31_ and S_32_, respectively), and reflection from port 3 (S_33_).

Considering all the geometrical details, a full-wave electromagnetic simulation preliminarily verified this configuration. The results of the simulation, when the switch at port 3 was considered as not actuated, are plotted in Figure 3, in terms of the matching at the input port (S_11_), transmission to the actuated branch (S_21_), and the isolation between port 1 and 3, and between port 2 and 3, respectively, S_31_ and S_32_. The reflections at the input port were below −20 dB until 25 GHz, then slowly degraded, but were still acceptable up to 30 GHz (<−15 dB).

Regarding the transmission to port 2, in this case, the chosen active branch of the device, represented by the S_21_ parameter, showed an insertion loss above −1 dB. Finally, the isolation between the disconnected ports, parameters S_31_ and S_32_, was from −30 dB to −20 dB at the upper band, a rather good value expected for the isolation, since it was obtained using only one not actuated switch.

**Figure 4:**
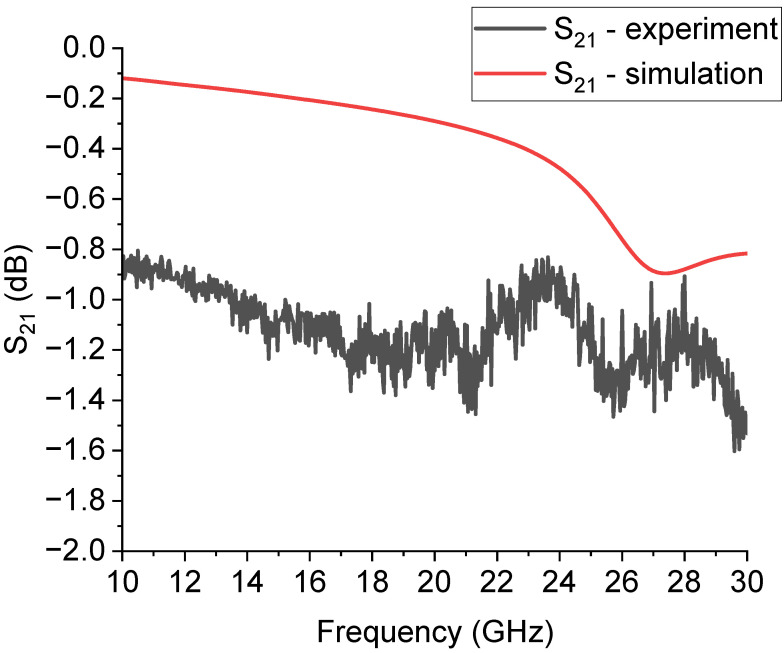
Comparison between the expected and the experimental S_21_ parameter, in dB scale, for the SPDT configuration used in this work. The losses are underestimated, especially below 30 GHz. On the other hand, an acceptable loss level, in the order of −1 dB, is obtained for the entire device transmission performance, comparable with the same configurations studied in the literature.

On the other hand, a complete analysis of this device should include a deformed bridge, electromechanically simulated to give a more realistic result. At the same time, most of the simulations in the literature have estimated the electrical response with a rigid ON–OFF functionality, moving the entire beam up or down with respect to the contact position. This gives back a normally underestimated performance depending on the operative frequency, because parasitic contributions cannot be correctly predicted. The situation is clarified in Figure 4, where the simulation is compared with the experimental result for the transmission parameter S_21._ The parasitic effects in the electrical response of the MEMS SPDT and the CPW lines were considered, including the material properties and thickness in the electromagnetic simulations. The dielectric loss contributions and the transmission lines’ losses are defined in terms of tanδ and bulk resistivity for the materials and the resistivity of the thick metal used to simulate the CPW lines. Of course, as discussed above, a complete analysis of the parasitics should include the deformed shape of the bridge and not simply a comparison between the up and down state as a rigid metal beam, but, in most cases, this approximation is valid for obtaining a reasonable agreement between a simulation and experiment [26]. Accounting for additional details is probably more critical for miniaturized RF MEMS switches, like those designed for higher frequencies [27]. Concerning the mechanical response and its latency, the MEMS devices were not minded for fast switching, but for providing response times in the order of tens of μs down to 1 μs, depending on the actuation procedure and the mechanical structure, and their utilization in redundancy and reconfiguration applications does not imply a fast electronic solution since the beginning [28].

**Figure 5:**
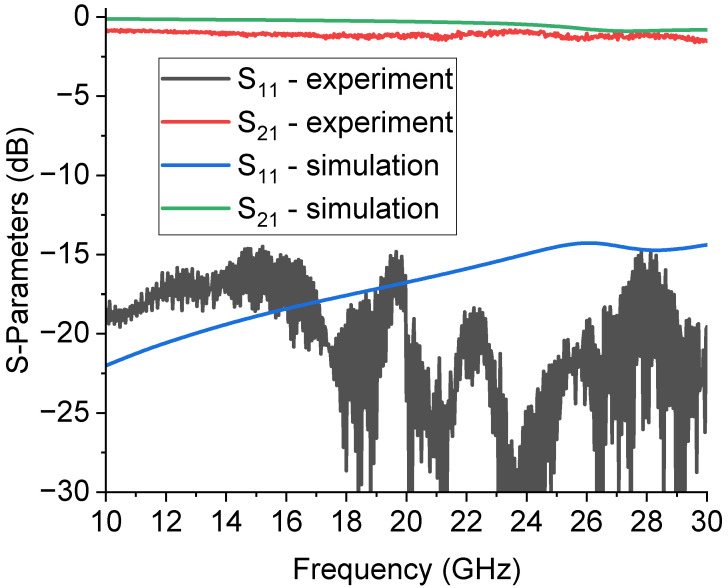
Experimental response of the RF MEMS SPDT. Reflection (S_11_) and transmission (S_21_) response, with port 3 in isolation.

Good reflection and insertion loss performances were obtained from an experimental point of view, as shown in the plot exhibiting the S_11_ and S_21_ parameters of the measured SPDT device in Figure 5, compared to the simulation. As expected from an ohmic switch, a wideband response was also achieved. Some boundary disturbances affected the measurement, which was noisy, still maintaining an acceptable value of the reflection coefficient S_11_ that was comparable to the expected values. In addition, multiple reflections caused notches in the S_11_ response, and this could be a sign that, when the switch was actuated, the non-flat metal beam was affected by local discontinuities that were not entirely considered. Other factors influencing the reflection could have been the right-angle, non-optimized CPWs transmitting the signal towards the resonators.

The actuation voltage for the first actuation was high, close to 80–90 V, but was reduced to 60–65 V after exercising the switch with more actuations, also contributing to removing some minor residuals that could still be present after the etching of the sacrificial layer.

## 3. Sierpinski Resonators

Triangular Sierpinski resonators were considered for their advantages in tuning capability by fixing the footprint of the resonator and increasing the internal complexity, i.e., changing the number of sub-triangles starting from the original full metal patch. In addition, it is easy to combine them in multi-resonant structures, coupling a few resonators in non-conventional geometries. As anticipated in the introduction, it is also interesting to study this geometry from a fundamental point of view, because a detailed theory of the resonant frequencies considering the CPW feeding of the resonators is not yet available for microwave signal processing. The resonators were fully embedded in a CPW boundary, and it is worth noting that this structure, as well as the U-shaped resonators discussed in the next section, is part of the more general family of metamaterial or fractal geometries corresponding to the additional properties in signal propagation [29,30]. They are characterized by the exhibition of an effective dielectric constant and a magnetic permeability that are both negative, contributing to backward radiation for antennas and significant changes in the propagation properties for planar components. Results and comments about these properties in the studied geometries can be found in [24], or in more general literature such as [25,31,32].

In our work, we preliminarily studied a few triangular Sierpinski resonators and selected the configurations promising for filtering adjacent frequencies for fine-tuning. Starting from the full triangular patch, we arrived at the third level of internal complexity determined by the number of sub-triangles, and we defined them as C0, C1, C2, and C3. Single resonator performances are not necessarily the best choice when combining two or more geometries into a complex one. For this reason, after considering the results of the single resonators, we manufactured and tested, for immediate purposes, a structure including the C0 and C1 triangles, i.e., the entire patch and the first level of complexity for the Sierpinski structure working at 20 GHz. At the same time, the C3 and the C2C3 mixed configurations were studied for the 26 GHz device. When we say “C2C3”, one of the triangles followed the C2 complexity, while the mirrored triangle was designed with the C3 geometry. The mirrored resonators in CPW configurations must have symmetric structures along the central conductor of the CPW and boundaries close to the ground to improve the electrical matching conditions. Primary considerations about the coupling issues and the spectrum reconstruction for CPW-fed Sierpinski structures were discussed in [24].

**Figure 6:**
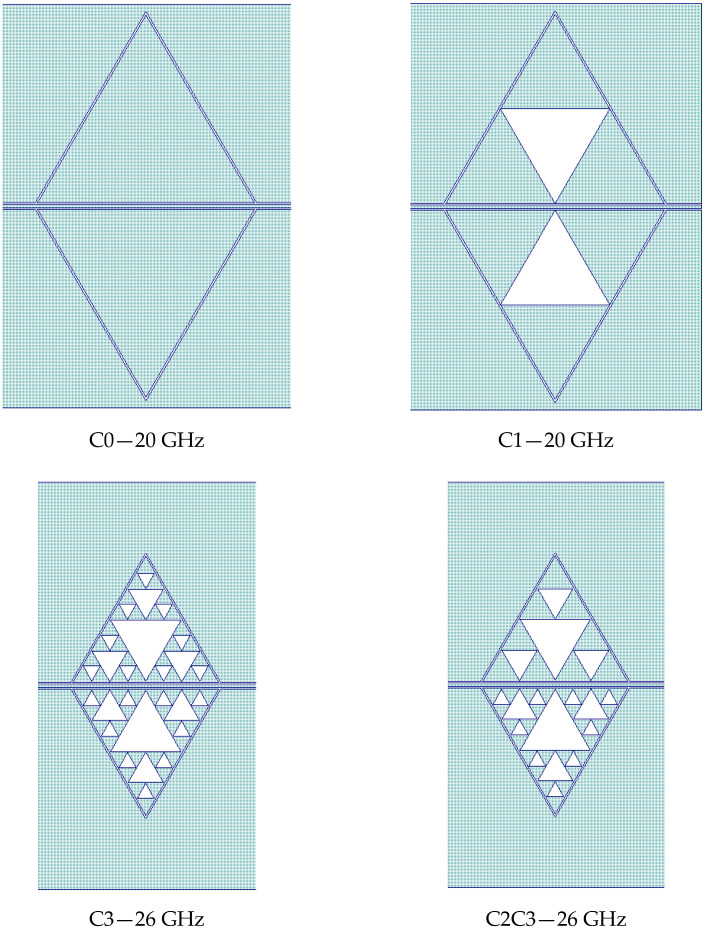
Resonator configurations are used for the 20 and 26 GHz operations. The length of the edge of the individual triangles is 4 mm for the 26 GHz and 6 mm for the 20 GHz operation, respectively. It is worth noting that the configuration C2C3 for 26 GHz is mixed, with the upper part obtained with the internal complexity “C2”, while the lower triangle follows the “C3” complexity.

All the used configurations, including the mixed one, namely C2C3 for 26 GHz, are represented in Figure 6. The difference in size was due to the two different working frequencies for the devices studied.

The individual response of the resonators was already studied in [24]. The approximated value of the wanted resonance frequency was obtained, accounting for the electromagnetic and experimental trials. Analytical tools are inadequate for predicting resonance frequency, because the theory found in the literature was developed for via-hole or microstrip-fed antenna structures. The CPW environment embedded the resonators completely, and this required a more complicated approach to derive the expected frequency of the resonance analytically for the triangular patch and Sierpinski triangles obtained with empty sub-triangles. So far, resonance frequencies close to the desired ones were obtained with the help of electromagnetic simulations and experimental verifications. In this case, the lengths of the edges for the triangles were 4 mm and 6 mm, respectively, corresponding to resonances around 26 GHz and 20 GHz approx.

### 3.1. Results on the Switched Triangular 20 GHz Resonators

**Figure 7:**
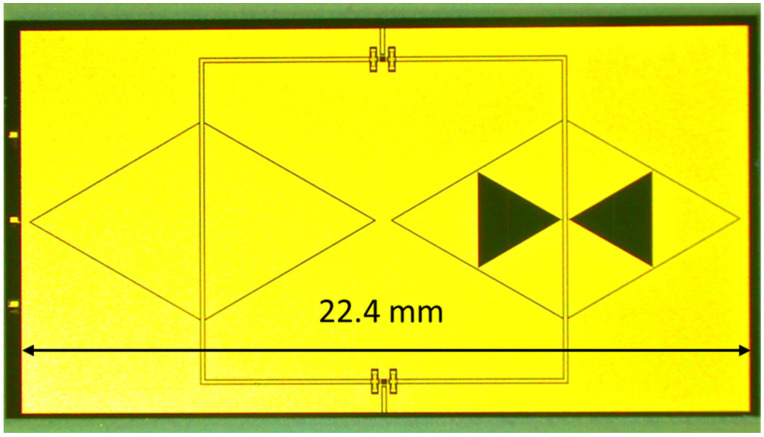
Layout of the switched C0 and C1 resonators for the 20 GHz operation. The RF MEMS switches are evidenced on the upper and bottom sides of the figure to allow the signal to pass through the C0 or the C1 resonator, depending on the actuated branch.

**Figure 8:**
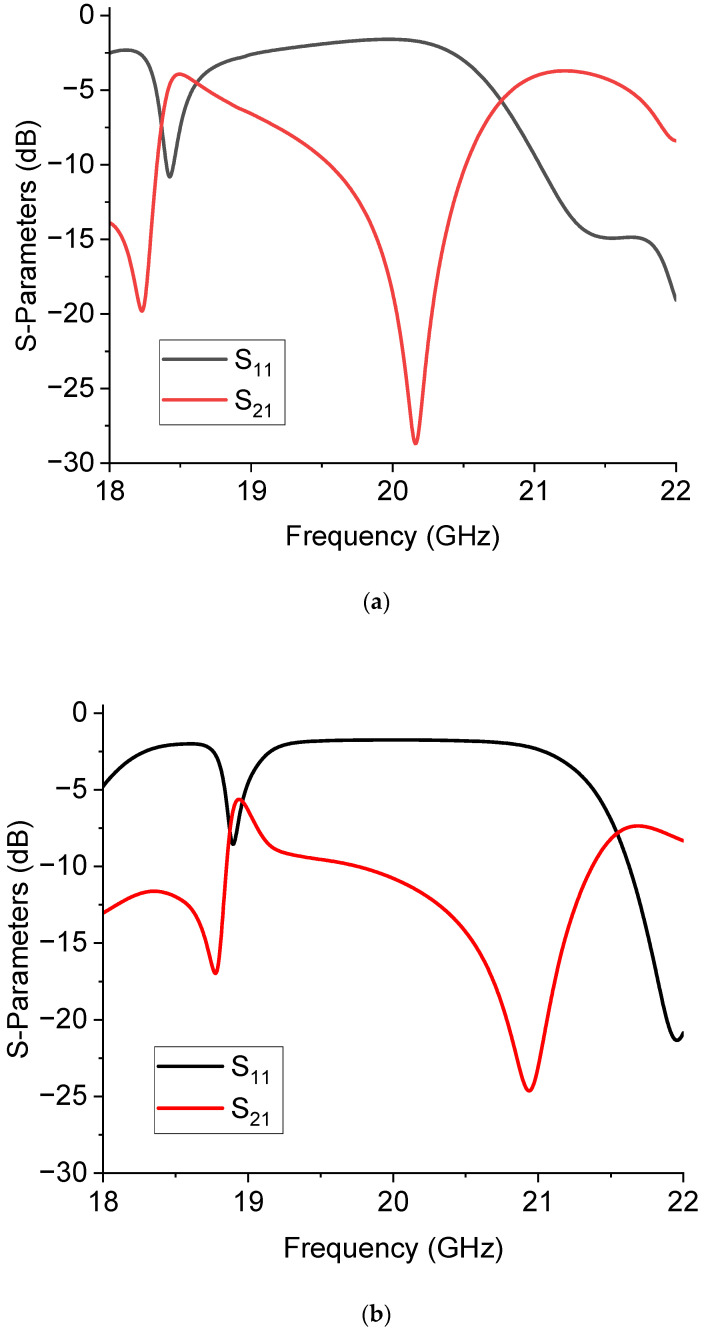
Simulation of the two-port filter implemented with the resonators C0 and C1 for the 20 GHz operation. In (**a**) the C0 configuration is activated, while in (**b**) the C1 resonator is used as the output of the SPDT-driven structure.

The results of the 20 GHz resonators, combining the C0 and C1 geometries, are presented in this section. The switched configuration is shown in Figure 7, where the two possible states are given, considering an actuation on the filter’s upper or lower branch, as shown in the figure. This configuration was simulated to predict its response in the two possible states, and the simulation is presented in Figure 8.

**Figure 9:**
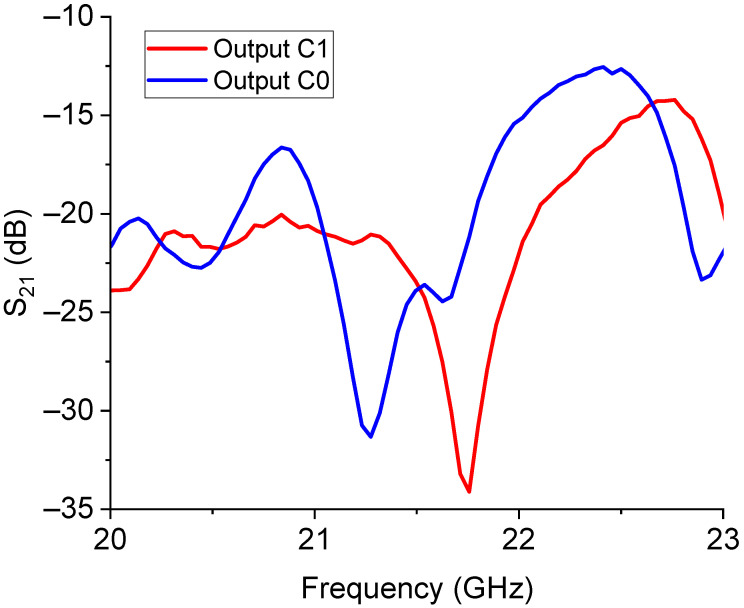
Experimental response for the 20 GHz switched filter with the C0 and C1 triangles.

The experimental result for the transmission S_21_ parameter of this structure is shown in Figure 9.

Despite the non-perfect matching causing significant losses along the line, the result agreed with the general expectations of this device, with a shift of the output resonance when the signal was switched from the C0 branch to the C1 one and a dynamic response for the ON/OFF states of the switch in the order of at least 15 dB. The predicted resonance frequency was around 20.16 GHz for the C0 response, while the measured one was 21.27 GHz; for the C1 output, the expected resonance was 20.93 GHz, while the measured one was 21.75 GHz. So, both frequencies were shifted by approx. 1 GHz, and the difference between the two frequencies of resonance was 500 MHz, and not 700 MHz as expected.

From the analysis of the obtained results, we can conclude that the big resonators working around 20 GHz probably interacted between them in proximity to the internal corners. Better isolation could be provided by a post of absorbing material separating the two devices transversally or shifting the relative position to avoid interference due to the proximity between the corners of the triangles. The situation also needed to be improved at the I/O ports of the entire device to decrease the insertion losses significantly. So far, proof-of-concept was demonstrated at this stage, but the complete device needed to be correctly engineered for application purposes.

### 3.2. Design and Test of 26 GHz Switched Triangular Resonators

**Figure 10:**
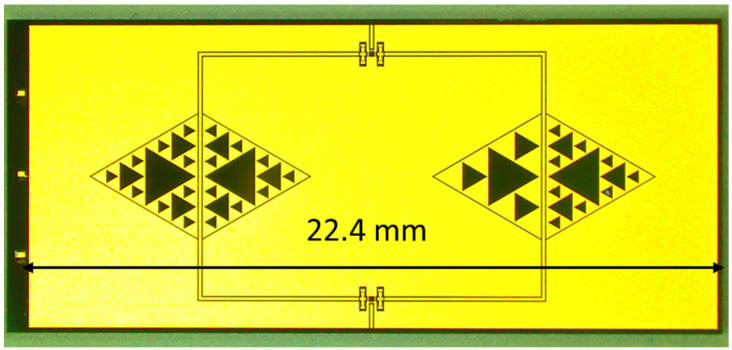
C3-C2C3 switched-resonator for the 26 GHz operation. The same criterion is used with respect to the 20 GHz resonators, with the SPDT driving the output signal through the C3 (left side) or the C2C3 (right side) structure, depending on the actuated branch.

The nominal 26 GHz switched resonator configuration is shown in the layout of Figure 10. In this case, a C3 configuration represents the left branch of the filter, while the C2C3 structure is on the right side.

**Figure 11:**
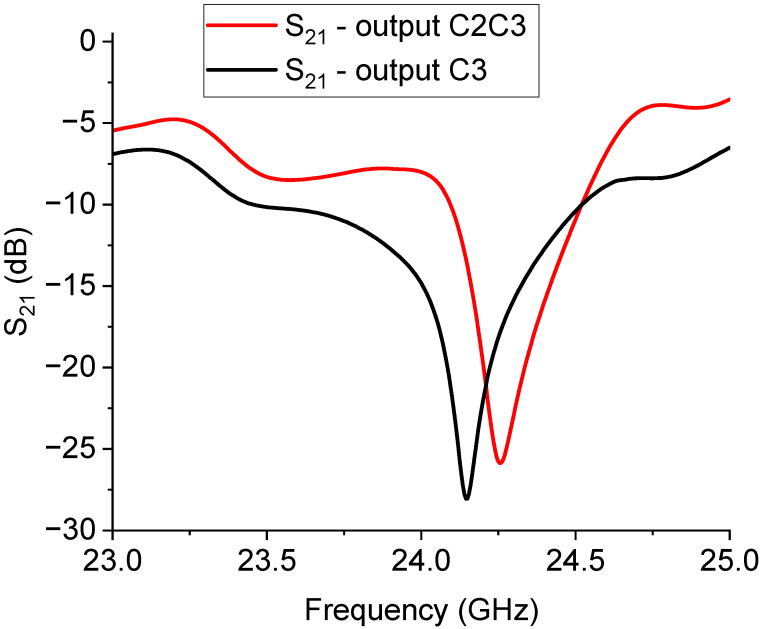
Simulation of the C2C3 structure for the 26 GHz filtering.

**Figure 12:**
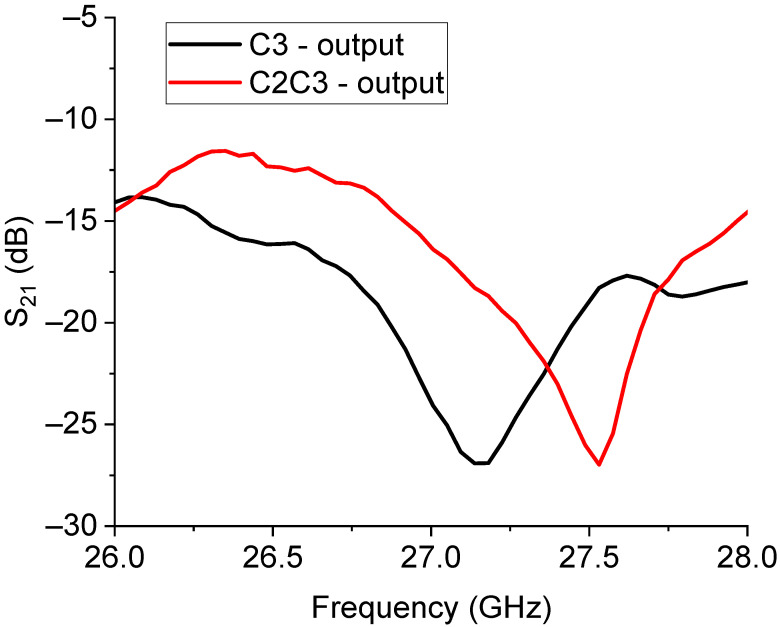
Measure of the 26 GHz filter performance.

The expected response for the 26 GHz filtering is shown in Figure 11, while the measured performance is given in Figure 12.

Looking at the experimental results, like in the case of the switched 20 GHz triangles, it is worth noting the necessity of improving the I/O coupling for engineering purposes. On the other hand, a dynamic ON/OFF response of the primary mode was obtained with approximately 15 dB of rejection. Nevertheless, a frequency shift of the main mode up to approx. 27 GHz had to be accounted for. In detail, the expected resonances for the entire structure were 24.14 GHz for the C3 output and 24.25 GHz for the C2C3 output, while the experimental responses were 27.13 GHz and 27.53 GHz for the C3 and C2C3 outputs, respectively. It must be stressed that the design was strongly affected by a shift with respect to the chosen resonance frequency, and this was particularly evident when the wanted resonance frequency was higher, beginning to be closer to the millimeter wave range. Such an effect was probably due to the necessity of an improved simulation effort on the entire structure, involving more geometrical details and a specific characterization of the employed materials, which could contribute to the equivalent circuit modifying the spectrum in a non-negligible way. While the preliminarily studied single resonators exhibited resonance frequencies closer to the expected, the entire structure, including the SPDT and the feeding lines and discontinuities (such as the bending on the corners), showed significant differences compared to the original design goals. In this case, smaller resonators helped to induce a decreased interaction, even if, also for this configuration, a shift in the position of the resonators could be helpful for more effective isolation between the two states.

## 4. U-Shaped Resonators

Until now, U-shaped resonators have been studied mainly in microstrip configurations for filtering applications or combined in arrays for antenna and high-frequency absorber purposes. A U-resonator alone is not necessarily suitable for a metamaterial response, at least the resonators studied in [24]. Still, their collective excitation is usually referred to as a metamaterial-inspired structure. The main advantage of a U-shape in filtering applications is the possibility of bending a resonating line with a consequent decrease in the footprint. This solution allows for a designer to use the U-elements in the well-established coupled-line filters designed in a microstrip configuration, substituting straight lines with bent ones. Preliminary work on filters implemented by RF MEMS switches was performed in [33]. The hairpin filter is a well-known example of using U-bended lines [34,35]. The drawback of the line-bending procedure is to account, during the design of the device, for the discontinuity created by the bending itself, whose equivalent circuit modifies the resonant frequency. Another delicate point to be considered is the coupling between the resonator and the feeding line, either a microstrip or a CPW.

**Figure 13:**
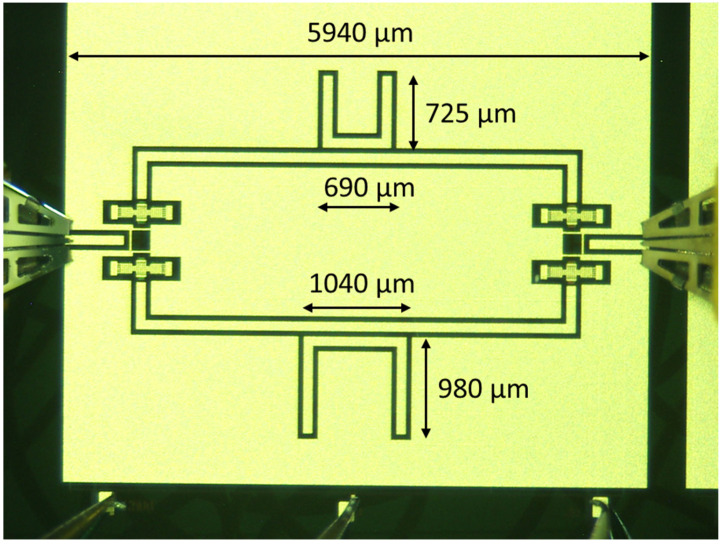
Layout of the U-switched resonators. In this picture, the upper resonator allows the 26 GHz output, while the 20 GHz resonator is shown on the bottom side. The size of the full structure and detailed dimensions of the single resonators are also given.

The structures studied in this paper combined a 20 GHz resonator and a 26 GHz resonator coupled to the central conductor of a CPW and were placed on two branches separated by an SPDT RF MEMS switch. The SPDT drove the signal on the branch selected by the actuation of the corresponding membrane. In the following Figure 13, the configuration used for the double-frequency filter is shown during an on-wafer measurement.

**Figure 14:**
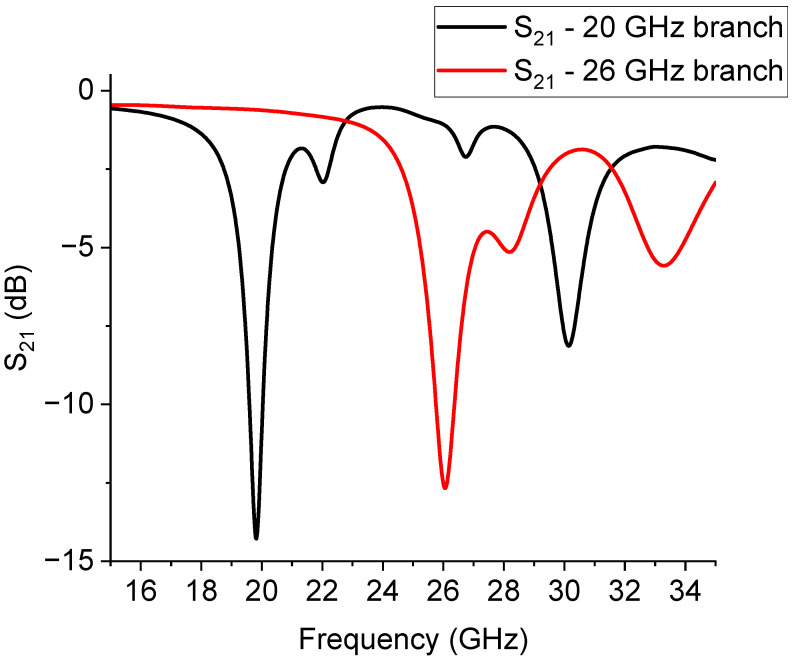
Expected resonances for the structure with two U-shaped resonators working at 20 GHz and 26 GHz, respectively.

In Figure 14, the predicted performance of the switched filter based on the U-resonators is shown, where the selected frequencies around 20 GHz and 26 GHz resulted from the simulation of the individual filter response. As previously discussed, additional resonances can appear in the spectrum, but having a series of resonances is intrinsic to the nature of all resonators, depending upon the size of the resonators, and corrected by their effective lengths because of electromagnetic boundary effects and the coupling conditions. The main mode was almost correctly predicted at 20 GHz; the same happened for the 26 GHz resonator.

**Figure 15:**
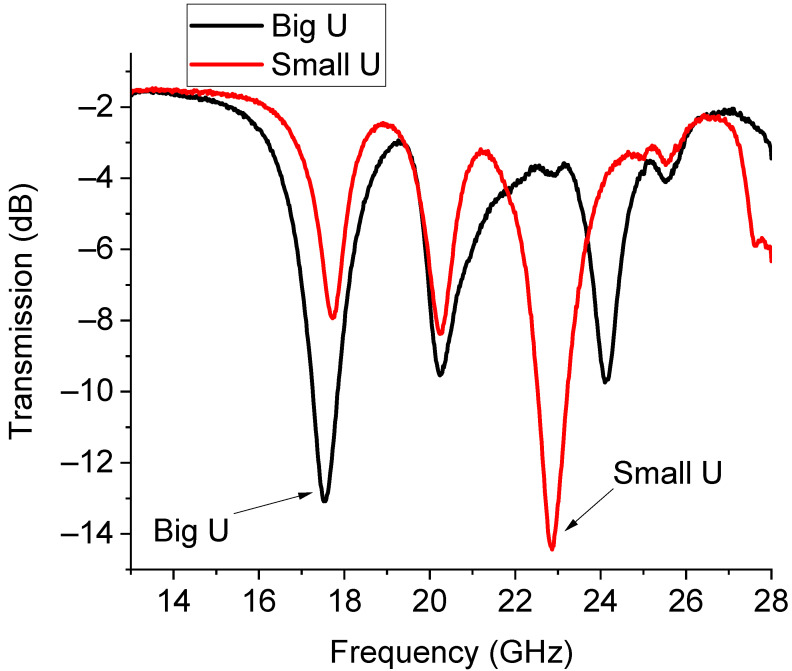
Resonance response for the switched U-resonators. Multi-resonant behavior is recorded. Evidence is given for the activation of the big and small resonators, originally designed for the 20 GHz and 26 GHz operations.

**Figure 16:**
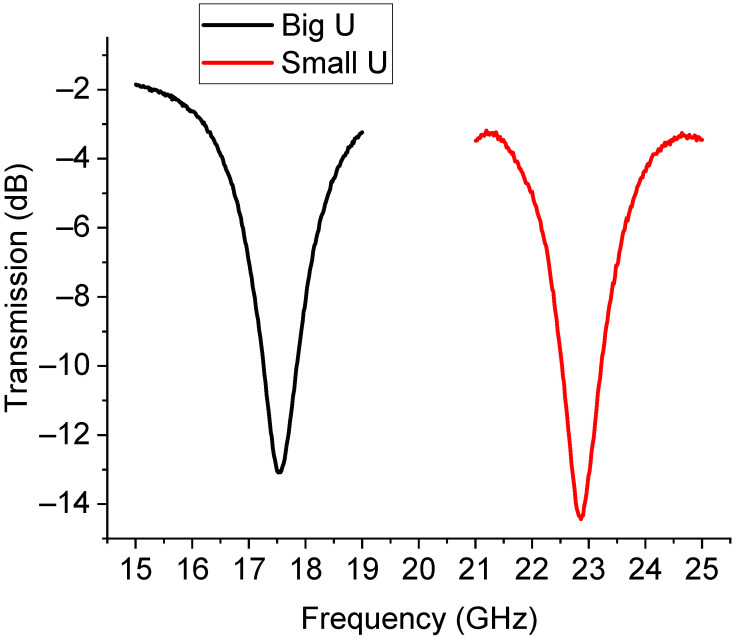
Detail of the main resonance for the big and small U-resonators. A significant shift is obtained with respect to the individual filters.

Experimentally, the situation was more complicated. The presence of the switches and the feeding lines, together with the intrinsic nature of the resonators of being multi-resonant, allowed for the excitation of multiple peaks because non-perfect matching or unwanted inhomogeneities created discontinuities that could excite higher-order modes, thus hindering the possible selection of two single resonances. This situation is clearly shown in Figure 15, where the measured response of the U-structures is plotted. An arrow is used to indicate the main resonance due to the single resonator (big or small) activated by the branch selected using the SPDT. Like in the case of the already discussed triangles, a further improvement of this configuration should include a shift in the position of the two individual resonators to provide a better physical separation and mitigate the possible interaction between them. Additionally, both the main resonance modes were shifted with respect to the expected single resonances because of the influence of the entire network. For clarity, the main modes were isolated from the previous figure and are plotted separately in Figure 16.

In Figure 16, the almost full correctness in the expected difference between the frequency of resonance of the small resonator and the big one is highlighted, even if the spectrum is shifted downwards. The expected frequencies for both resonators were 19.8 GHz and 26.02 GHz (from Figure 14), while the experimental ones were 17.52 GHz and 22.82 GHz (from Figure 15). So far, a downward shift of approx. 2 GHz was experienced when comparing the theory and experiment.

In conclusion, the studied resonators are promising as individual devices. Still, a better selection is needed, accounting for the multi-resonant response of the entire structure and the shift induced by the influence of all the circuital components.

## 5. Conclusions

This paper proposed switched triangular and U-shaped band-stop resonators implemented by MEMS technology, employing an RF MEMS SPDT to select the path for the RF output. The individual resonators were: (i) Sierpinski-based triangular structures obtained by increasing the internal complexity of a full metal patch using empty internal sub-triangles and (ii) U-shaped structures. Both switched resonators were designed in CPW configurations and are suitable for more complex structures involving coupled individual resonators. Devices working at 20 GHz and 26 GHz approx. were studied for potential K-Band operation, i.e., in the band presently more appealing for satellite applications, proving quite good performances and suitable optimizations. Promising results were obtained for both the resonator configurations, although some optimizations are still needed to improve the electrical matching of the entire structure. The SPDT exhibited a good performance in terms of the insertion losses, with a measured value for the transmission parameter S_21_ of around −1 dB in a wide frequency range, thus confirming the wideband capabilities of ohmic RF MEMS switches. A further engineering step is necessary for improving the insertion losses of the full devices, keeping control of the parasitic contributions and discontinuities. On the other hand, the basic requirements for selecting two different frequencies were demonstrated by a rejection ratio of nearly 15 dB between the ON/OFF states of the switches. Furthermore, additional work is needed to predict the resonance frequency better when a high complexity of the Sierpinski configuration is used.

## Data Availability

Raw data available upon requirement.
